# How Does Collective Moral Judgment Induce Unethical Pro-Organizational Behaviors in Infrastructure Construction Projects: The Mediating Role of Machiavellianism

**DOI:** 10.3390/bs13010057

**Published:** 2023-01-08

**Authors:** Qinqin Xiong, Qi Pan, Shangyao Nie, Fei Guan, Xinyu Nie, Zhoubao Sun

**Affiliations:** 1 School of Engineering Audit, Nanjing Audit University, 86 West Yushan Road, Nanjing 211815, China; 2 Nanjing Audit Bureau, 18 Beijing East Road, Nanjing 210000, China; 3 Antai College of Economics and Management, Shanghai Jiao Tong University, 1954 Huashan Road, Shanghai 200030, China; 4 Jiangsu Key Laboratory of Public Project Audit, Nanjing Audit University, 86 West Yushan Road, Nanjing 211815, China

**Keywords:** sustainable development, unethical pro-organizational behavior, collective moral judgement focus on self, Machiavellianism, performance-avoidance goal orientation, motivation to learn

## Abstract

The sustainable development of infrastructure construction projects heavily depends on favorable cooperation of all parties and ethical code of conduct, while Un-ethical pro-organizational behavior (UPB) may undermine the mutual efforts and cause serious consequences. UPB has aroused wide interest of researchers, but what may trigger construction employees to engage in UPB at team-level has not been elucidated completely. With information asymmetry and huge uncertainty, the behaviors of employees in temporary project teams are marked by environmental and personal characters. The study discusses the influences of collective moral judgement focus on self (CMJS) and Machiavellianism on UPB. Through a moderated mediation analysis conducted on a set of survey data from Chinese construction projects, the empirical results of the two-level hierarchical linear model indicate that CMJS positively impacts UPB directly, and meanwhile Machiavellianism acts as a partial mediator in the relationship between CMJS and UPB. The findings also reveal that performance-avoidance goal orientation (PAGO) and motivation to learn (MTL) moderate and strengthen the relationship between Machiavellianism and UPB. The study offers practical suggestions for both project managers and policymakers of construction projects.

## 1. Introduction

Since unethical pro-organizational behavior (UPB) was conceptualized and theorized nearly ten years ago, it has aroused wide interest from researchers to explore UPB phenomenon [1,2,3,4,5,6]. Different from apparent unethical behaviors for self-serving, UPB refers to unethical behaviors which individuals engage in for the interests of their organizations and members voluntarily [1]. Scholars believe UPB is widespread in workplace [7]. Organizations may benefit from UPB for the moment. But, potentially, despite the “good” intentions, UPB often induces bad consequences [8,9,10]. And the same is true in infrastructure construction projects which are generally significant and concerned with public welfare. Infrastructure construction projects are unique with integrated fragment structure involving various professionals working together [11,12,13] with fierce competition and ethical dilemmas [13,14]. With one-off organizational form and tasks of complex uncertainty and variability [15,16], construction employees have to play a proactive role and work flexibly to get project objectives. And meanwhile, the common situations of asymmetric information and imperfect contracts between different stakeholders in construction industry urge people more likely to break moral codes to serve their temporary teams (for instance, the voluntary efforts to conceal project environmental violations or cover up construction quality problems, etc.). Some studies have explored that unethical behaviors (including unethical behaviors for self- interest and UPB) may cut across all stages (Project selection, Planning, Inspection, Design, Bid and contract signing, Construction, Service delivery, Maintenance) of construction projects globally [11,17,18,19,20], such as falsification of reports, deliberate concealment errors, etc. Perceived as for a higher purpose than personal greed, UPB is more disguised and deceptive. As infrastructure construction project objectives heavily depend on favorable cooperation of all parties and ethical code of conduct [21,22], at the expense of the interests of stakeholders outside, UPB in infrastructure construction projects will increase conflicts of interests and reputational risks to cost and time overruns [11], even cause serious consequences, such as safety accidents, quality defects which may induce fatalities [14], or reduce effective social services and public goods [17] as the result of projects failure. The morally questionable events and behaviors in infrastructure construction projects call for broad concerns.

Researchers have achieved some remarkable results based on empirical methods. The studies have identified some key factors and the ensuing psychological processes through which employees engage in UPB, and indicated some boundary conditions as well. The dominant perspectives obtained from individual, supervisor and organizational levels on the antecedents include: positive reciprocity, organizational identity, organizational commit, ethical leadership, etc. [7,8,23,24,25,26,27], and some of which also been perceived as boundary conditions (such as social identity, positive reciprocity, etc.) [1,5,6,24,26].

Although these literatures have revealed some precursors of UPB in workplace, the studies mainly focus on stable and relatively permanent organizations, whose polices, cultures and employees’ attitudes towards the organizations (for example, organizational commitment) have formed and played roles for a certain period, which may not clarify UPB in temporary project teams. In the uncertain and dynamically complex situations, as well as the external constraints of balancing the interests of different stakeholders [28], employees in infrastructure construction projects act with greater autonomy and flexibility to match the changing environment, that also makes team members’ behaviors marked by environmental and individuals’ characteristics. For temporary organizations, regulations can only provide general guidelines for action, and construction contracts are results-oriented (such as time limit, quality index, budgets, etc.), it is beyond formal rules of project teams to regulate employees’ behaviors. In such circumstances, informal rules, routines, practical experiences and person-situation interactions play a greater role [29]. When employees confront moral dilemmas, the ethical work climates they share in teams are the dominant moral perceptions, and are valid indicators of ethical behaviors of members [30,31]. Collective moral judgement (CMJ) provides employees moral reasoning norms to judge whether their pro-organizational behaviors are morally right. The current findings about the relationship between CMJ and ethical behaviors in stable organizations are mixed [32]. Given the importance of informal regulations and person-situation interactions to temporary organizations and the members, CMJ might affect employees’ moral behaviors in infrastructure construction projects, but there has been little research.

To address these questions, this paper tries to explore how and when CMJ affect UPB in temporary organizations. Based on social cognitive theory, CMJ shapes project employees’ moral cognition, which naturally influences one’s moral behaviors. With the collective moral judgement focus on self (CMJS), construction employees may form the psychological cognition that self-interest is the main concern, which drives individuals to engage in UPB. Organizational context, meanwhile, may impact personality traits. Some studies have showed the links between workplaces and the personality traits, Machiavellianism involved [33,34]. Machiavellianism shows the propensity to manipulate others in pursuit of selfish gains [35]. Scholars found the possibility that ethical work climates may shape Machiavellian’s behaviors [29]. CMJS encourages employees to act self-centred, to some extent, the ethical work climate provides the moral justification for employees to pursue their own interests while breaking through inherent moral rules [36,37]. Under the constraints of time, resources and interests of infrastructure construction projects, team members work together for the projects while competing with each other and feeling threatened by colleagues and implicit social comparisons. It may stimulate the desire for status and control and the tendency to manipulate others ignoring moral considerations and further conduct people to engage in UPB. Additionally, drawing on trait activation theory [38], environments vary in the degree to which they motivate the expression of trait relevant behavior [39], we argue that some psychological processes held in individuals’ seeking performance may have some unclear impact on the relationship between Machiavellianism and UPB. On the path to project success, the critical performance metrics of projects generally are decomposed into individuals and project teams certainly will continuously evaluate and feedback the stuff to achieve the expectations. Employees with different goal orientations differ in their reactions and behaviors [40], such as individuals with performance avoidance goal orientation (PAGO) may appear threatened and resist, or rise to the occasion [41]. They can act for collective gains as to contribute to their own’s performances [7]. With high PAGO, the employees feel great pressure of performance which may motivate the Machiavellians hold strong intentions to engage in UPB. The performance pressure and innovative tasks also makes that employees’ prior knowledge and skills may not be sufficient to cope with the uncertainty and complexity in temporary teams, and learning at work is considered normal. The desire to learn in workplace is positively related to skill acquisition [42] which may help ones to get ahead as they desire. Such tendencies as goal orientation and motivation to learn (MTL) reshape the psychological environment which may exert an influence upon UPB. To contribute insight into the occurrence of UPB in temporary teams, this study explores the effect of CMJS on UPB and the internal mechanisms by a moderated mediation analysis, based on a cross-level analysis through a questionnaire survey of 777 project employees nested within 137 infrastructure construction project teams. The empirical results show that CMJS positively effects UPB and Machiavellianism plays an intermediary role between CMJS and UPB while PAGO and MTL positively moderate the mediating effect.

## 2. Theoretical Background and Research Hypotheses

### 2.1. Social Cognitive Theory

Social cognitive theory (SCT) expounds the individual’s cognitive process that standards and norms can be delivered to individuals through social learning to form the cognition which can shape individuals’ behaviors [43]. In SCT, with certain organizational context, behavioral agents act in compliance with work ethical standards through regulating self-psychological mechanism (including self-efficacy and response-outcome expectancy). Especially, the theory emphasizes the interaction and correlation between social environment, internal personal features (including cognitive system) and behavior patterns.

SCT has been valuable in previous empirical research on UPB [9,24,44]. Drawing on the theory, the process by which the moral norms are dismissed and masked is perceived as a cognitive process, while unethical behaviors occur as a result. With certain social context and/or personal factors, such as political work environment [44], driven by teams’ interests, employees may acquire some new moral cognition and then engage in UPB. In this study, we take the theory as the theoretical base to clarify the mechanism of occurrence of UPB.

### 2.2. Unethical Pro-Organizational Behavior of Infrastructure Construction Projects

The concept of UPB is defined as “actions that are intended to promote the effective functioning of the organization or its members (e.g., leaders) and violate core societal values, mores, laws, or standards of proper conduct” [1]. It emphasizes two core conceptual components: first, UPB is unethical as it violates ethical standards and guidelines; second, UPB is pro-organizational behavior, with the intension to benefit organization or its members, acted voluntarily.

Absorbing all connotations of UPB defined by Umphress and Bingham [1], the “organization” refers to infrastructure construction project teams in this study. Project is one-off while project teams are temporary organizations. Project team generally is composed of knowledge employees from different professional background and technical expertise to achieve project objectives [45]. There are instances where multiple project teams working simultaneously in one construction enterprise. Therefore, UPB in the study represents unethical behaviors voluntarily intended to benefit the project team or its members. From the perspective, project team and(or) its members benefit from UPB, to some extent, while the organization involving the project team may be one of the outside stakeholders and harmed.

UPB in infrastructure construction project act mainly in two ways: seeking profit and avoiding loss. Driven by urgent project performance requirements, project employees put a lot of efforts into maximizing teams’ interests proactively when make decisions and actions. UPB is often the case in seeking profit. Such as, unprompted jerry-build in concealed works. In other cases, with asymmetric information, individuals adopt an active pose which may violate moral norms, through evading risks or concealing project facts, to protect the interests of projects or the members [46]. For instance, confronted with the risk of fines for delays, employees choose to meet tight deadlines late in night with noise, disregarding the surrounding residents; or buck-passing of engineering accident voluntarily. UPB in infrastructure construction projects benefiting teams in short terms may disrupt collaborative relationships between project stakeholders and cause serious consequences for the stealth, deserving more attention.

### 2.3. CMJS and UPB

According to Victor and Cullen [47], ethical work climate presents the dominant ethical perceptions of collective employees, impacting the decision and action that employees take when faced with moral dilemmas. Perceived as a psychological process of dealing with ethical issues, moral judgement refers to shared moral reasoning and the norms of what action is morally right [22]. Essentially, moral judgement is a kind of moral cognition, which reflects individuals’ understanding of moral issues and guides their moral behaviors [48,49]. Reynolds et al. further presented that moral judgement was the main cognitive motivation for unethical behavior [50]. In team level, CMJ involves the collective form of moral reasoning that guides team members which behavior is morally right, including CMJ with focus on self (CMJS) and CMJ with focus on others [22]. CMJS is the moral view in an egoistic moral context that one’s own interests dominate others.

According to cognitive theory, CMJS mainly triggers UPB from two aspects. On one hand, the behavior patterns of employees in CMJS are influenced by the self-interested climates and motivated to meet the collective norms [43,48,51]. CMJS identifies the particular “self” in whose interests one is expected to act. With high level of CMJS, individual forms new moral cognition that seeking one own’s interest is morally right and acts in compliance with the collective norms through observing self-interested behaviors that often occur and social learning [52]. In infrastructure construction projects, team member’s own welfare largely depends on project earnings [6]. The self-interested context of CMJS sends clear signals that team members maximize the interests of internal stakeholders is morally right and the criterion of success, while little considerations for outside ones [53,54,55]. The fact that individual’s own interests and collective interests are deeply inter-twined makes individuals earn through seeking collective interests as far as possible [56], including unethical behaviors. Meanwhile, CMJS may limit individuals’ moral knowledge [6,57]. As CMJS is strong, when faced with the moral dilemma of conflict of interests inside and outside the project team, employees are unwilling to consider the complex ethical implications of their behaviors [58]. As such, employees may be incline engage in UPB to help project teams as well as benefit themselves.

On the other hand, CMJS plays an important role to maintain psychological security for the team members who engage in UPB. Generally, organizations set framework for employees’ ethical behaviors through norms that support ethical behaviors in workplace [54,58]. In temporary teams, CMJS signals that employees are allowed to pursue their own’s interests, disregarding the concerns of outside stakeholders [59]. CMJS provide a psychological safety climate which has an impact on one’s attitudes and behaviors for the employees where they feel confident to act regardless of the consequences without fear of punishment or retribution [6,60,61]. With the moral climate team members may even perceive that UPB is morally right. And the perceived ethicality of UPB will further promote the occurrence of UPB [6,62,63].

As such, we propose that:

**Hypothesis** **1:** *Collective moral judgement focus on self positively influences unethical pro-organizational behavior*.

### 2.4. The Mediating Role of Machiavellianism

Machiavellianism in managerial context describes the tendency of an individual to pursue one’s self-interests by manipulative and ruthless means. It was conceptualized in four personal attributes: *amoral manipulation*, *distrust of others*, *desire for control* and *desire for status* [64]. Researchers generally think Machiavellians disregard morality for their purposes. Although it is known for the value orientation “ends justify the means” [65], the very start point of Machiavellianism is to meet their own desires.

According to SCT, influenced by the team-level ethical work climate, employees’ original moral schema may be constantly assimilated by the collective ethics. Individual’s own moral structure is standardized by the shared perceptions to keep to the team, through which the moral judgement may be the core of individual’s sense of self [66]. Accordingly, self-centered inner power increasingly internalizes to individual’s enduring trait which may drive employees to do anything, even at the expense of others, to satisfy their own needs. Employees may use various means to achieve their goals, such as political skills which positively relate to Machiavellianism [64], and have a negative view of human nature, with treating co-workers as instrumental men who just served for their goals. In the pursuit of maximizing their own interests, it’s possible that they perceive threatened by others or external risks, being in a strong position will be a good option. It can be argued that, to some extent, the internal belief and motivation of CMJS coincides with high Machiavellianism. Meanwhile, disregarding standards of traditional morality is core and prerequisite to Machiavellianism [64]. CMJS provides supportive atmosphere to motivates employees to chase their self-serving goals and more tolerance for unethical behaviors. The climate provides psychological security for Machiavellians.

In recent researches Machiavellianism has been confirmed to be positively correlated with unethical behavior [19,67]. And Machiavellians show strong economic opportunism in moral behaviors [68]. Despite perceived as “dark side” in management [69], Machiavellians may do “good things” for their teams. The “utilitarian sense of morality” [7] which acts directly on subconscious decisions may drive them to engage in everything to satisfy their own self-interests. If the team’s goals align with individual goals, or achieving individual’s aim depends on the team objectives, Machiavellians may tend to engage in pro-organizational behaviors, whether moral or not. Moreover, reciprocity beliefs may drive Machiavellians engage in UPB [70]. If they predict that UPB will promote the interests of return, Machiavellians will do. The reciprocal exchange of interests prompts Machiavellians to form transactional psychological contracts with their teams [71] and increases the willing to engage in UPB.

Therefore, it is proposed that:

**Hypothesis** **2:** *Machiavellianism mediates the positive relationships between collective moral judgement focus on self and unethical pro-organizational behavior*.

### 2.5. Moderating Effect of Performance-Avoidance Goal Orientation

Trait motivation theory is a person-situation interaction approach which proposes that relevant organizational situations may trigger the behaviors related to personal traits in workplace [72,73]. Prior findings have suggested that there is a direct link between Machiavellianism and unethical behaviors [7,48,50,51]. In temporary teams, personal interests are largely consistent with team interests, Machiavellians may engage in unethical behaviors that may serve for their groups [7]. As long as they stand to benefit from UPB, Machiavellians hold strong intentions to act.

Performance-avoidance goal orientation (PAGO) describes the desire to avoid exposure of one’s incompetence and to avoid negative comments on one’s ability [33]. Employees with PAGO are conditioned to choose conservative task strategies to avoid be criticized. They are more aware of potential risks and more likely to flinch in the face of challenges [55]. With the performance pressures, infrastructure construction projects highlight their output objectives which conduct periodic and irregular evaluations of employees’ performance. In the climate of CMJS, coworkers pursue their own and teams’ goals, contributing to the competitive work environments which are perceived by individuals with PAGO [74]. Individuals with PAGO may perceive enormous stress of demonstrating low ability. With the passive psychological experience and negatives situational cognition, Machiavellians acquire the psychological climate perception that avoiding disproving one’s competence is acknowledged as the most important aspect in construction projects while ethics can be disregarded [75,76]. For simplicity, we call the individual climate perception as PAGO-climate perception here.

As Machiavellianism predicts unethical behaviors [7,67], drawing on trait motivation theory, PAGO-climate perception increases the relationships between Machiavellianism and unethical behaviors [77,78]. As personal interests mainly are from teams’ interests, PAGO-climate perception augments the relationships between Machiavellianism and UPB. And when the propensity of PAGO is higher, individual’s PAGO-climate perception will be stronger, and Machiavellians’ willingness to engage in UPB will be stronger.

Therefore, it is proposed that:

**Hypothesis** **3:** *Performance avoidance goal orientation positively moderates the mediating effect of Machiavellianism on the relationships between collective moral judgement focus on self and unethical pro-organizational behavior*.

### 2.6. Moderating Effect of Motivation to Learn

Motivation to learn (MTL) describes individuals’ willingness to learn and improve their own capacities. MTL is an effective way to improve oneself. Individuals high in Machiavellianism attempt to be dominant and powerful [65]. However, not every Machiavellian can get ahead as they desire. Whether Machiavellians are successful to some extent depends on their skills including technical skills and social skills. Technical skills are undoubtedly necessary to achieve goals, while social skills are critical as well. Social skills are crucial workplace-specific social competency [72], also called political skills [73]. As the propensity of individuals high in Machiavellianism (such as to distrust others, manipulate, seek status, and to have instrumental relationships with coworkers) and undesirable behaviors in workplace may trigger poor reputation and broken partnerships which will impede Machiavellians’ efforts to their desires [79]. High degree of political skills can clear the way. Political skills cover four important aspects [80,81,82]: social astuteness, interpersonal influence, networking ability, and apparent sincerity which help individuals act appropriately along with changing situations to obtain project resources. Meanwhile, Machiavellians with great political skills can plan and adopt complex tactics to maintain trust, cooperation, good reputations, that will play the protective role, through which individuals can reduce the risk of exposure their real intensions.

Individuals with MTL are willing to learn and improve the abilities to deal complex situations. For their own self-serving purpose, Machiavellians with high MTL will effectively capture the knowledge and abilities that they need to gain their goals and develop their political skills as much as possible. And then, the stronger of MTL Machiavellians have, the more political skills and other skills they possess, consequently, the willingness and competence of Machiavellians engaging in UPB will be stronger.

Therefore, it is proposed that:

**Hypothesis** **4:** *Motivation to learn positively moderates the mediating effect of Machiavellianism on the relationships between collective moral judgement focus on self and unethical pro-organizational behavior*.

**Figure 1:**
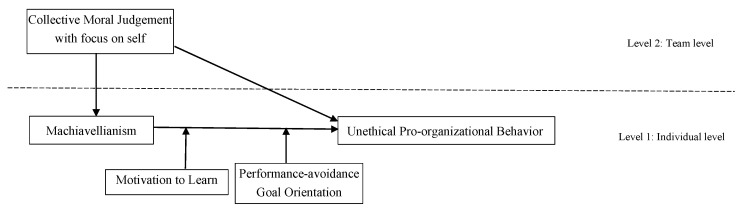
The conceptual model of the research.

The conceptual model is presented in Figure 1.

## 3. Methodology

### 3.1. Sample and Data Collection

The study adopted a set of questionnaire survey data from Chinese infrastructure construction enterprises. The survey used a two-way translation by two experienced scholars in project management research and revised the scales cautiously to avoid culture bias and ensure validity. Moreover, we interviewed experienced scholars and practitioners and conducted a pretest to verify the content, clarity, and phraseology of the items before the formal investigation. Integrating the feedback and suggestions, the questionnaire consisted of two parts. One project manager completed Part 1 that contained independent variables related to projects. Several project staff in the same project were required to fill Part 2 containing dependent variables, mediating variables, and moderating variables which were related to individual level. Questionnaires are completed anonymously and it was clearly stated that there were no right or wrong options [18]. We asked the China Association of Construction enterprises (CACE) to select some firms as our object samples. We distributed two hundred sets of questionnaires on-site and got 137 sets completed questionnaires including 48 firms, 137 project teams and 777 project individuals. In the sample, the mean of team size is 15.74 members (SD = 15.27); the mean of project age is 4.74 years (SD = 3.52). The average working years of the respondents is 4.60 years (SD = 4.98). The average working years of the respondents in the company is 5.55 years (SD = 4.87).

### 3.2. Measurements

All the perceptual items were designed on seven-point Likert scales, and the average scores were calculated for every multi-item construct.

#### 3.2.1. Dependent Variable

Unethical Pro-Organizational Behavior. UPB reflects the unethical behaviors for project team’s or its members’ interests here. To measure UPB, we adopt a six-item scale from Umpress [8]. Sample items include “I would distort the truth to make my project look good if it would help my project team” and “If it would benefit my team, I would overstate my project’s products or services to customers”. The Cronbach’s *α* for UPB is 0.89.

#### 3.2.2. Independent Variable

Collective moral judgement with focus on self. CMJS brings one’s moral decision-making framework. To measure CMJS, the study follows Arnaud [32] and adopts the five items to assess the propensity of the respondents. The example items are: “In the project team, people put their own interests above other considerations”, “People in the project team are very concerned about what is best for them personally”, “The employees in the project team are mainly for their own benefit”, etc. Its Cronbach’s *α* is 0.86.

#### 3.2.3. Mediator

Machiavellianism. In line with Dahling [64], Machiavellianism is measured from four dimension: desire for status, distrust for others, desire for control and amoral manipulation. The set of sixteen items include three measuring desires for status, five representing distrusts for others, three showing desires for control, and five for amoral manipulation. Cronbach’s alpha coefficients for Machiavellianism was 0.82.

#### 3.2.4. Moderator

Performance-avoidance goal orientation (PAGO). Consistent with Don Vandewalle [33], a five-item scale is used to measure PAGO. The sample items are “If this matter will show that I appear inferior to others, I would avoid taking it” and “Avoiding displays of low competence is more important than learning something new”. Its Cronbach’s *α* is 0.82.

Motivation to learn. MTL describes employees’ desire to learn in the workplace [83]. Consistent with Yu [42], MTL is measured with a four-item scale. The example items are “I am motivated to learn the skills emphasized in the project”, “I try to learn as much as I can from my project”, etc. Its Cronbach’s *α* is 0.87.

#### 3.2.5. Control Variables

Based on the available literature, five demographics variables are controlled to avoid the potential effects. Gender is a dummy variable (1 = “male”, 0 = “female”). Age is expressed by integer values from 1 to 4 (1 = “below 29”, 2 = “30 to 34”, 3 = “35 to 39”, 4 = “40 years old and above”). His/her education experience (Edu) is represented by numeric variables (1 = “high school”, 2 = “a junior college degree”, 3 = “a bachelor degree”, 4 = “a master degree”, 5 = “a doctor degree”). Company tenure (CT) and project tenure (PT) are self-reported in years.

### 3.3. Confirmatory Factor Analysis

This study tests the construct validity of the multi-item scales through a confirmatory analysis. All the Average Variance Extracted (AVE) after getting the Cronbach’sαof the latent variables (>0.7), they are greater than 0.5, and the root mean square of AVE of the variable is greater than the coefficients of correlation between the variable and other variables. It means the convergent validity and discriminant validity of the variables meet the requirement. We present five alternative models including 3 four-factor models, 1 three-factor model and 1 one-factor model, compared with the baseline model, and the results are shown in Table 1. Comparing with the other five models, the baseline model shows the best fitting degree with the sample data: *χ^2^/df* = 2.076, IFI = 0.971, TLI = 0.964, CFI = 0.971, RMSEA = 0.037, SRMR = 0.037. All the values are within acceptable ranges and obviously better than which of other models. These results reveal that the construct validity of the measure model in this study is acceptable.

## 4. Results

Table 2 describes the mean, standard deviation of each variable in this study, and shows the Pearson correlation coefficients between the variables, all of which are less than 0.75, within acceptable scope.

A two-level hierarchical linear modeling (HLM) is used to analyze individual level UPB which is nested in team level. CMJS which reflects the common form of moral reasoning in the social system refers to team level. HLM is used to assess relationships of variables within and between hierarchical levels simultaneously, to estimate the effect of factors at different levels on outcome variables at the individual level, and to improve the estimation of individual-level effects. In this study, the intra class correlation(1) (ICC(1) = 0.3454), the intra class correlation(2)(ICC(2) = 0.7495), with-group agreement (r_wg_ = 0.915) meet the requirement of reliability. The regression result is shown in Table 3.

**Figure 2:**
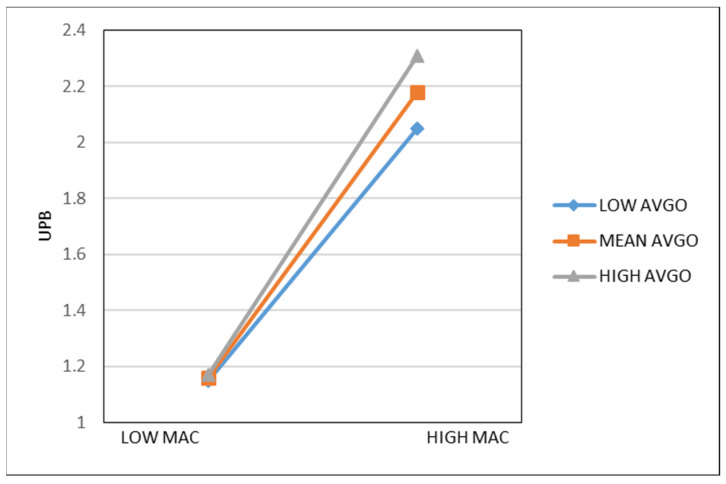
The Moderating role of PAGO on the relationship between MAC and UPB. Note: UPB = Unethical Pro-organizational Behavior; MAC = Machiavellianism; PAGO = Performance-avoidance Goal Orientation.

**Figure 3:**
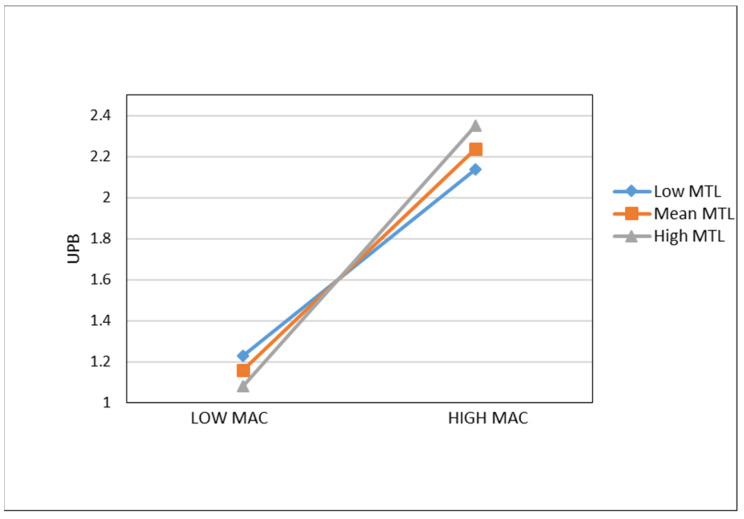
The Moderating role of MTL on the relationship between MAC and UPB. Note: UPB = Unethical Pro-organizational Behavior; MAC = Machiavellianism; MTL = Motivation to Learn.

Table 3 summarizes the results of the analysis. Model 1 and Model 3 are the regression analyses of Machiavellianism and UPB by the control variables, to control the influence to the dependent variables. Model 4 shows the regression analysis of CMJS to UPB, indicating CMJS positively influences UPB (β = 0.129, *p* < 0.05), which supports H1. It means CMJS (with focus on self) shared by project teams will induce the members to engage in UPB. Model 2 shows that CMJS positively affects Machiavellianism (β = 0.139, *p* < 0.01). Model 5 incorporates both CMJS and Machiavellianism into the regression analysis of UPB. It is found that CMJS is significantly positively related to UPB (β = 0.127, *p* < 0.05) with controlling Machiavellianism and the regression coefficient of CMJS to UPB (β = 0.127) is less than the coefficient which in Model 4 (β = 0.129), meanwhile, the influence of Machiavellianism on UPB is significant (β = 0.612, *p* < 0.001). The results shows that Machiavellianism plays a partial mediation role between CMJS and UPB, and H2 is testified. Generally, infrastructure construction project employees are under dual leadership: one from project teams, other from the original organizations or departments whose rules play a limited role in one-off projects. Team contexts, such as the conceptions, procedures, work climate shared in projects teams, have a more important and direct influence. CMJS in project teams encourages individuals to pursue one’s own interests whether its moral or not; meanwhile, CMJS reshapes one’s original moral schema and strengthen one’s self-centered power to Machiavellianism and further affects UPB through Machiavellianism. The moderate effects which are proposed in H3 and H4 are tested in models 7–10. In order to reduce the potential multicollinearity between variables in the regression equations, this study constructs the interaction terms by mean-centralized the mediator and moderator. The results of Models 7 and 8 shows that the direct influence of PAGO on UPB is not significant (β = 0.037, *p* > 0.05 for Model 7, β = 0.043, *p* > 0.05 for Model 8), but PAGO significantly positively moderates the influence of Machiavellianism on UPB (β = 0.083, *p* < 0.05). The relationship between Machiavellianism and UPB is also positively moderated by MTL (β = 0.179, *p* < 0.01), although the direct effect of MTL on UPB was not significant (β = −0.057, *p* > 0.05 for Model 9, β = −0.058, *p* > 0.05 for Model). We conduct a Bootstrap procedure to examine the moderated mediation effect. The results represent 95% confidence interval doesn’t contain zero. As Table 4 shows, the indirect effects of CMJS on UPB through Machiavellianism vary with MTL/PAGO at different levels (−1 SD, Mean, +1 SD). When the condition of MTL/PAGO is high (vs. low), the indirect effect increases. Thus, the findings support H1–4. The relationships between Machiavellianism and UPB are plotted under the case of high and low level of PAGO in Figure 2. It shows the relationship between Machiavellianism and UPB is strengthened when PAGO is high. And Figure 3 reveals MTL increase the relationship between Machiavellianism and UPB when MTL is as predicted in H3 and H4.

## 5. Discussion and Implications

Unethical Pro-organizational behaviors in the field of infrastructure construction project management have attracted much attention and criticism. Compared with routine management, project is marked by one-off and complexity which makes project behaviors strong personal vision [84], and the unethical behaviors in the name of the project team are more destructive and hidden. To answer the urgent need, this study explores the driving factors of UPB in project teams through a cross-level empirical research. Enlightened by the proposal that moral behaviors of individuals are influenced by the ethical work climate, of which the most important component is CMJ [32,47], the study theoretically proposes that UPB of project employees may be positively affected by CMJS. The study probes the mechanisms by which CMJS affects UPB of project individuals, and the conditions in which UPB occurs are also discussed.

The empirical results reveal that, as expected, CMJS influences significantly UPB. Specifically, with CMJS, the project employees are inclined to engage in UPB. Meanwhile, Machiavellianism partially mediates the relationship between CMJS and UPB. CMJS promotes the personal trait of Machiavellianism and further triggers UPB. In addition, we determine that the strength of the relationship of Machiavellianism and UPB is enhanced at high levels of PAGO in certain organizational contexts, MTL as well. These findings offer not only new theoretical understandings, but also valuable managerial implications for infrastructure construction project management.

### 5.1. Theoretical Contributions

This study responses the call to carry out researches on team-level antecedents of UPB [9], and advances the literature on UPB in team contexts. First, it provides new clues on driving factors regarding ethical work climates in project teams. This study explores the cognition, behaviors, and psychological adjustment of project employees, as well as the interaction between individuals and the ethical work climates, and finds that CMJS positively affects UPB in construction projects. CMJS identifies the particular “Self” in whose interests one is expected to act. It shows that, the shared aggregate perceptions, norms, attitudes have an important impact on temporary organizations, which also indicates that the strength of the team cohesion alone is not enough, it is also necessary that the cohesion is in line with the team’s values. Also, it may provide a new perspective for further exploration of the consequences of UPB: UPB may benefit or harm teams, where teams also weigh the interests and loss. As CMJS conducts employees’ behaviors through reshaping one’s moral cognition, it is viable for teams to guide clear expectations for moral behaviors by creating the appropriate ethical work climates.

Second, this study identifies the important driving mechanism and contextual conditions of UPB. The study examines the channels by which team’s ethical work climate influences individual’s psychological characters and then translates into action. The propensity of CMJS improves individuals’ characteristics of Machiavellianism, while Machiavellianism tends to unethical behaviors which benefit both oneself and the project teams. The moderated mediation analysis indicates the interaction between personal psychological characters and personal social-cognitive contexts involving APGO and MTL would impact the occurrence of UPB. We find that high APGO increase the effect of Machiavellianism influencing UPB, as well as high MTL. MTL is considered to promote positive outcomes [42,83,85], while we find the interaction between Machiavellianism and MTL will impact UPB. Machiavellians with high MTL will master more political skills and other skills that help them engage in UPB more subtly and effectively.

Furthermore, our research provides a broader understanding of UPB. According to the definition of UPB [1,8], the motivation of employees engaging in UPB is to benefit organizations or their members. Many antecedents of UPB in previous research, such as organizational identity, positive reciprocity beliefs, organizational commitment, ethical leadership, show pro-organization tendency [5,26,86,87]. While both CMJS and Machiavellianism convey self-interested purposes, this study further indicates that UPB may be not completely independent of self-interested motivation, more likely, employees engage in UPB with the combination of two motives.

### 5.2. Managerial Implications

These findings also provide practical suggestions for infrastructure construction project managers and decision makers on controlling UPB. As the empirical results of this study reveal the impacts of CMJS and personal propensity of Machiavellianism on UPB, it may be an effective approach for projects to prevent UPB from both ethical work climates and individual’s traits. On the one hand, project teams should have a broader vision and normalize the form of moral reasoning with focusing both self and others. A straightforward approach is to pay more attention to the interests of other stakeholders, not just self-interests, nourishing good morality at organizational level which project team members come from. On the other hand, project managers should concern about personal attributes in project and identify the individuals with the propensity of Machiavellianism who in key positions are likely to engage in UPB. It is managers’ responsibility to match job and person properly and effectively to avoid UPB in advance. Moreover, the assessment of employees should be more based on the growth and development of employees, rather than focusing on current performance. The project individuals and teams should hold learning goal orientation.

Policymakers should highlight the improvement of ethical work climates and industrial assessment system. On the one hand, it may be workable to establish institutional ethics rating system for infrastructure construction companies through specific ethical indicators and norms to assess organizational ethical levels, as to lead the construction industry to pay attention to moral system. On the other hand, the industry may strengthen the assessment of projects from the sustainable development and the social long-term goals replacing current project performance, especially for infrastructure construction projects. And the interest of other stakeholders of projects, such as the public, should be taken into the value in the process of assessment.

## 6. Limitations and Future Research

First, our cross-sectional design only describes one point in time and can’t show the potential causal relationship, while the mediating effects partly explain the causation in the study. Future research may adopt multisource data and a longitudinal design to catch the dynamic changes. Also, one possible flaw comes from self-assessment response which may be overly positive. Instead of measuring actual UPB, we assessed the willingness of the action. Nonetheless, studies found there were little differences between intentions and actual unethical behaviors [7,67]. The measurement of actual UPB should be considered in the future. Moreover, the measurement scales adopted in this study are kept with existing western designed scales which may not be adequately capture the nuances of the variables examined in Chinese culture. UPB may have more abundant connotations in Chinese collectivist cultural background and individuals may be more prone to engage in UPB for collective interests. Future research may seek to develop indigenous scales to verify the relationships in different cultures.

## 7. Conclusions

This study demonstrates whether and how CMJ affect UPB in infrastructure construction projects. CMJS induces UPB. As CMJS shapes individual’ moral cognition, Machiavellianism proposes an internal mechanism that links CMJS in team level and construction employees’ UPB. MTL and PAGO, as the contingent factors, strengthen the relationships of CMJS-Machiavellianism-UPB. The findings prompt it is viable to conduct appropriate ethical work climate for temporary organizations to control UPB.

## Figures and Tables

**Table 1:** Comparison of Measurement Models.

Model	χ^2^	df	χ^2^/df	Δχ^2^/Δdf	IFI	TLI	CFI	RMSEA	SRMR
Baseline model	226.249	109	2.076	--	0.971	0.964	0.971	0.037	0.037
Four factors (MTL + CMJS)	436.642	113	3.864	190.807	0.921	0.904	0.920	0.061	0.060
Four factors (CMJS + PAGO)	389.793	113	3.449	555.478	0.932	0.918	0.932	0.056	0.054
Four factors (PAGO + UPB)	290.441	113	2.964	321.317	0.952	0.941	0.952	0.050	0.047
Three factors (MTL + CMJS + PAGO)	580.051	116	5.000	391.792	0.887	0.866	0.886	0.072	0.070
One factors	2734.242	119	22.977	478.497	0.360	0.266	0.358	0.168	0.116

Note: CMJS = Collective Moral Judgement with focus on self; UPB = Unethical Pro-organizational Behavior; MTL = Motivation to Learn; PAGO = Performance-avoidance Goal Orientation.

**Table 2:** Descriptive Statistics and Pearson Correlation Matrix.

	Mean	S.D.	Age	Gender	Edu	PT	CT	CMJS	MAC	UPB	MTL	PAGO
Age	1.159	0.366	1									
Gender	3.037	1.265	−0.118 **	1								
Edu	2.590	0.690	0.054	−0.010	1							
PT	4.119	4.734	−0.013	0.329 **	0.126 **	1						
CT	6.365	5.093	−0.057	0.640 **	0.007	0.322 **	1					
CMJS	2.153	0.978	0.000	−0.047	0.122 **	0.106 *	−0.090 *	1				
MAC	2.060	0.797	−0.031	0.069	0.079 *	−0.253 **	0.056	0.167 **	1			
UPB	1.685	0.836	−0.029	−0.013	0.107 **	−0.176 **	−0.012	0.176 **	0.652 **	1		
MTL	4.278	0.737	−0.056	−0.030	−0.041	−0.053	0.012	−0.183 **	−0.173 **	−0.087 *	1	
PAGO	3.162	0.977	−0.035	−0.099 **	−0.076 *	−0.156 **	−0.042	0.071 *	0.217 **	0.211 **	0.150 **	1

Note(s): Significance levels: ** *p* < 0.01, * *p* < 0.05; CMJS = Collective Moral Judgement with focus on self; UPB = Unethical Pro-organizational Behavior; MAC = Machiavellianism; MTL = Motivation to Learn; PAGO = Performance-avoidance Goal Orientation; Edu = education; PT = project tenure; CT = company tenure.

**Table 3:** Regression results.

Variable	MAC	MAC	UPB	UPB	UPB	UPB	UPB	UPB	UPB	UPB
	Model 1	Model 2	Model 3	Model 4	Model 5	Model 6	Model 7	Model 8	Model 9	Model 10
CMJS		0.139 **		0.129 *	0.127 *					
MAC					0.612 ***	0.613 ***	0.722 ***	0.692 ***	0.728 ***	0.727 ***
PAGO							0.037	0.043		
MTL									−0.057	−0.058
MAC*PAGO								0.083 *		
MAC*MTL										0.179 **
Age	−0.287 **	−0.285 **	−0.171	−0.170	0.006	0.005	−0.008	0.017	−0.014	0.023
Gender	−0.006	0.000	−0.050	−0.045	−0.036	−0.039	−0.038	−0.035	−0.042	−0.034
Edu	0.156 **	0.152 **	0.140 *	0.136 *	0.051	0.054	0.050	0.032	0.034	0.027
PT	−0.027 **	−0.029 **	−0.012	−0.014	−0.001	0.000	0.002	0.000	0.000	0.000
CT	0.019 *	0.019 *	0.011	0.012	0.001	0.001	−0.002	−0.003	−0.001	−0.003
Constant	1.725 ***	1.720 ***	1.538 ***	1.534 ***	1.746 ***	1.750 ***	0.118	1.766 ***	0.520	1.791 ***
*R* ^2^	0.286	0.270	0.357	0.345	0.395	0.395	0.445	0.447	0.445	0.452
Chi-square	576.764	554.835	522.073	509.687	749.144	749.144	542.000	541.000	542.000	541.000

Notes: Significance levels: *** *p* < 0.001, ** *p* < 0.01, * *p* < 0.05; CMJS = Collective Moral Judgement with focus on self; UPB = Unethical Pro-organizational Behavior; MAC = Machiavellianism; MTL = Motivation to Learn; PAGO = Performance-avoidance Goal Orientation; Edu = education; PT = project tenure; CT = company tenure.

**Table 4:** Moderated Mediating Effect of Machiavellianism by PAGO/MTL.

Moderator	Condition	Effect	SE	BootLLCI	BootULCI
**PAGO**	*Mean-1SD*	0.608	0.064	0.483	0.733
	*Mean*	0.692	0.041	0.610	0.773
	*Mean+1SD*	0.775	0.044	0.688	0.862
**MTL**	*Mean-1SD*	0.626	0.053	0.513	0.720
	*Mean*	0.727	0.038	0.653	0.801
	*Mean+1SD*	0.837	0.051	0.737	0.937

Note: The Bootstrap test is with 5000 resamples. CI = confidence interval; SE = standard error; MTL = Motivation to Learn; PAGO = Performance-avoidance Goal Orientation.

## Data Availability

To obtain the dataset analysed for this study, please contact xiongqinqin@nau.edu.cn or 270247@nau.edu.cn.
